# Resistify: A Novel NLR Classifier That Reveals Helitron-Associated NLR Expansion in Solanaceae

**DOI:** 10.1177/11779322241308944

**Published:** 2025-01-22

**Authors:** Moray Smith, John T Jones, Ingo Hein

**Affiliations:** 1 Cell and Molecular Sciences Department, The James Hutton Institute, Dundee, UK; 2 School of Biology, University of St Andrews, St Andrews, UK; 3 School of Life Sciences, University of Dundee, Dundee, UK

**Keywords:** NLRs, plant disease resistance, transposable elements, Helitrons, Solanum

## Abstract

Nucleotide-binding domain leucine-rich repeat (NLR) proteins are a key component of the plant innate immune system. In plant genomes, NLRs exhibit considerable presence/absence variation and sequence diversity. Recent advances in sequencing technologies have made the generation of high-quality novel plant genome assemblies considerably more straightforward. Accurately identifying NLRs from these genomes is a prerequisite for improving our understanding of NLRs and identifying novel sources of disease resistance. While several tools have been developed to predict NLRs, they are hampered by low accuracy, speed, and availability. Here, the NLR annotation tool Resistify is presented. Resistify is an easy-to-use, rapid, and accurate tool to identify and classify NLRs from protein sequences. Applying Resistify to the RefPlantNLR database demonstrates that it can correctly identify NLRs from a diverse range of species. Applying Resistify in combination with tools to identify transposable elements to a panel of Solanaceae genomes reveals a previously undescribed association between NLRs and Helitron transposable elements.

## Background

The plant innate immune system is an essential part of plant disease resistance. A key component of this is the nucleotide-binding domain leucine-rich repeat (NLR) proteins – an abundant and diverse family present in plant and animal genomes. The NLRs act as intracellular immune receptors which elicit an immune response after the detection of pathogen-derived factors or their activity.^1^ Since their discovery, hundreds of functional NLRs have been cloned and many sequences have been identified.^2^ The abundance of NLRs and their sequence diversity shows considerable variation across the plant kingdom.^3^

One driving force of NLR diversification is thought to be the proliferation of transposable elements (TEs). In *Arabidopsis*, sites of high TE insertion frequency have been previously identified to contain NLRs^4^ and NLRs exhibiting elevated intraspecific diversity are in close proximity to TEs.^5^ Of the TEs, long terminal repeat retrotransposons (LTRs) are the most prolific in plant genomes and are a major factor in genome expansion.^6^ Strikingly, the *Capsicum annuum* genome exhibits a marked expansion of NLRs nested within LTR-retrotransposons, and this effect is seen in other Solanaceae including tomato and potato.^7^ Other TEs, such as the Helitrons, can also capture and transport genes across the plant genome.^8^

Canonical NLRs have a modular organisation. Central to the NLR structure is the highly conserved NB-ARC domain which regulates the activity of the protein. Downstream of this is the leucine-rich repeat (LRR) domain which has a role in ligand binding and auto-inhibition. Upstream of the NB-ARC domain is variable. Often, there is a coiled-coil (CC), Resistance to Powdery Mildew 8-like (RPW8), or Toll/Interleukin-1 receptor/Resistance protein (TIR) domain. Together, this modular structure means that NLRs can be separated into different classifications: CNL, RNL, TNL, or NL if lacking a conserved N-terminal domain.

Recently, additional NLR-associated signatures have been identified. For example, the post-LRR C-terminal jelly-roll/Ig-like domain (C-JID)^9^ may be present in TNLs and appears to bind with pathogen effectors, thereby contributing to the immune response. Similarly, other NLRs have integrated decoys to detect pathogen activities. In terms of initiating cell death through the formation of a resistosome, CNLs rely on an N-terminal MADA motif, a hallmark of NRCs – NLRs which act as intermediaries in the NLR induced immune response or NLRs that function independently of NRCs.^10^

Due to their conservation and modular structure, NLRs lend themselves to automated identification and classification. To date, several tools have been developed to achieve this including DRAGO2, NLGenomeSweeper, NLR-Annotator, RGAugury, RRGPredictor, and NLRtracker (Table 1).^11–16^ The development of the RefPlantNLR database has allowed the benchmarking of these tools against the sequences of functionally characterised NLRs.^16^ Performance varied between tools with NLRtracker being the most sensitive and accurate. All tools perform well at the identification of TIR, RPW8, and NB-ARC domains which are highly conserved, but are often less accurate in predicting CC domains which are more variable and frequently missed by InterProScan.^17^ Additional to the currently available NLR classification tools is NLRexpress – a set of machine learning predictors for CC, TIR, NB-ARC, and LRR motifs.^18^ Although NLRexpress does not directly identify or classify NLRs, it is well suited for rapidly and accurately screening large sets of sequences for NLR-associated motifs. One strength of NLRexpress is the CC-associated extended EDVID model which performs strongly in identifying this challenging motif.

**Table 1.** A summary of the currently available NLR annotation tools.

Tool	Input data	Method	Output	Distribution
DRAGO2	Protein, Transcript	HMMER, COILS, TMHMM	Classification, Domains	Online only, API available
NLGenomeSweeper	Transcript, Genomic	InterProScan, MUSCLE, TransDecoder, BLAST, HMMER	Classification, Genome position, GFF annotation	GitHub, Manual dependency installation
NLR-Annotator	Transcript, Genomic	MEME^a^	Classification, Genome position, GFF annotation	GitHub
RGAugury	Transcript, Genomic	InterProScan, nCoils, pfam_scan, Phobius	Classification, Genome position, GFF annotation	GitHub, Online or local webservice, Docker container
RRGPredictor	Protein, Transcript	InterProScan	Classification	GitHub, Manual dependency installation
NLRtracker	Protein, Transcript	InterProScan, HMMER, MEME	Classification, NB-ARC sequence, domains, GFF annotation	GitHub, Manual dependency installation
NLRexpress	Protein	HMMER, Scikit-learn	Motif position, motif sequence	GitHub, Online or local, Conda environment provided
Resistify	Protein	HMMER, NLRexpress	Classification, NLR sequence, NB-ARC sequence, motif position, motif sequence	GitHub, PyPI, Conda, Docker, Singularity

Source: Adapted from Kourelis et al.^16^

a An additional version is available that does not rely on MEME.

One drawback with the currently available tools is the reliance on InterProScan as the backend for domain annotation. InterProScan is designed as a comprehensive and generalised domain annotation tool. As a result, each input sequence is scanned against several databases each containing in total more than 180 000 protein signatures, the vast majority of which are not NLR-associated. Compounding this, NLRs only represent a fraction of a plant proteome resulting in unnecessary searches against non-NLR sequences. In addition, domains common to NLRs are present in InterProScan databases with differing levels of curation which often results in overlapping or fragmented annotations. These must be parsed, particularly the LRR domain which is represented by multiple InterProScan signatures. The large databases that InterProScan relies upon must also be downloaded which provides an additional challenge for installation or automated deployment.

Software that is readily available with minimum manual configuration is a requirement for genome assembly and annotation projects. Projects may use an array of different tools, making manual installation and dependency resolution time-consuming. Dependency resolution solutions, such as Conda, and automated deployment of tools in pipelines with Snakemake or Nextflow are becoming increasingly common.^19,20^ In addition, bioinformatics research is often restricted to shared high-performance clusters (HPCs) where users have limited privileges and where storage is at a premium. Tools that require root privileges, are distributed as web services, or require large databases are often not conducive for genomics projects.

Here, the new NLR annotation tool Resistify is presented that overcomes some of the constraints of currently available tools. We show that this tool accurately predicts NLR sequences from diverse plant using a range of well curated sources and can be used for pangenome analysis of NLRs in Solanaceous genomes. In addition, we use Resistify in combination with the EDTA tool to investigate the genomic organisation of NLRs and their association with TEs.

## Methods

Resistify is implemented in python as a command line executable. First, Resistify executes an hmmsearch of input protein sequences against a custom database of HMMs derived from curated Pfam entries (Table 2). These models are used to identify CC, RPW8, TIR, and NB-ARC domains, as well as C-JID and MADA motifs. Domains of the same type are merged if they overlap or are within 100 amino acids of each other. Preliminary testing in development showed that this is necessary to overcome NB-ARC domain annotations which can become split. MADA hits with a score greater than 20 are considered genuine, whereas hits with a lower score are considered as MADA-like. Using this evidence, proteins are initially classified as belonging to a CN, RN, TN, or N classification. Proteins which do not have any evidence of an NB-ARC domain are discarded at this stage unless they are retained through –ultra mode.

**Table 2.** HMM models including in the initial hmmsearch stage of Resistify.

Article I. Domain	Article II. Source
NB-ARC	https://www.ebi.ac.uk/interpro/entry/pfam/PF00931/
TIR	https://www.ebi.ac.uk/interpro/entry/pfam/PF01582/
TIR	https://www.ebi.ac.uk/interpro/entry/pfam/PF13676/
RPW8	https://www.ebi.ac.uk/interpro/entry/pfam/PF05659/
CC	https://www.ebi.ac.uk/interpro/entry/pfam/PF18052/
C-JID	https://www.ebi.ac.uk/interpro/entry/pfam/PF20160/
MADA	https://cdn.elifesciences.org/articles/49956/elife-49956-supp2-v2.hmm

Following this, the filtered set of NB-ARC containing proteins is screened for NLR-associated motifs using a reimplementation of NLRexpress. Candidates are searched against the NLRexpress database with jackhmmer and the output profiles used as input for the NLRexpress classifiers. The jackhmmer search is parallelised for greater multithreading support. Sequences are then reclassified using the following logic. If a protein belongs to class N (ie, does not have any evidence of an upstream CC, RPW8, or TIR domain), it is scanned for upstream TIR or CC motifs. As motif searches can be more promiscuous, restricting motif searches to this condition prevents them from interfering with nonambiguous NLR classification. The sequence is then screened for LxLxxL motifs to define the LRR domain. Following a previous definition, an LRR domain is annotated if 4 or more LxLxxL motifs are identified with inter-motif gaps of less than 75 amino acids.^21^ Gaps larger than 75 amino acids are predicted to be a break in the LRR domain and so the LRR annotation process is restarted from that position onwards. If less than 4 motifs exist across the whole sequence, this process is skipped. Gap size and minimum motif count can be adjusted by the user.

This combined evidence is then integrated into the domain annotation data. The domains are sorted by start position and a ‘domain string’ is formed. For example, if the sorted domains took the order TIR, NB-ARC, LRR, C-JID, then the domain string would be TNLj. Alternatively, a canonical CNL with an MADA motif would take the form mCNL. The domain strings are searched for substrings CNL, RNL, TNL, or NL and classified accordingly. In addition, a count of the unique occurrences of the conserved NB-ARC motifs is made as an indicator of NB-ARC integrity. A ‘motif string’ is also created which represents the order of NLRexpress motifs across the sequence.

The primary output of Resistify is a table detailing the NLRs identified and the specific classification for each sequence. The complete motif string, domain string, classification, NB-ARC motif count, and MADA and CJID status are listed. A complete list of all annotations and NLRexpress motifs are given as separate tables. In addition, all NLRs and NB-ARC domain sequences are extracted and provided as FASTA files.

In some circumstances, such as screening a locus for putative resistance genes, it might be of interest to retain highly fragmented/partial NLRs which would be discarded by default. For this purpose, –ultra mode is provided which retains all sequences for the NLRexpress search and can identify sequences with, for example, a single LRR motif. As a result, sequences are not provided a classification.

Resistify is provided as a single executable available for download on GitHub, PyPI, and Conda. All databases and models are distributed with the executable so that manual setup is not required. All output files are placed in a nameable output directory for easy integration into automated workflows. Temporary files are also handled internally to reduce output clutter and improve performance. Resistify requires scikit-learn v0.24.2 and hmmer v ⩾ 3.0.

### RefPlantNLR benchmarking

Protein sequences of the RefPlantNLR database members were retrieved and used as input for Resistify with default settings.^16^ Resistify classifications were compared directly in R v4.3.2 with tidyverse v2.0.0. Any sequence where the Resistify classification did not exactly match with the provided RefPlantNLR structure was taken as a potential misclassification.

### Araport11 benchmarking

The latest release of Araport11 representative gene model protein sequences were downloaded from TAIR and used as input for Resistify with default settings.^22^ A phylogenetic tree was built from the Resistify-extracted NB-ARC domain sequences with mafft v7.52.0 and fasttree v2.1.11 with default settings.

### Pangenome pipeline

A Snakemake workflow was developed to predict genes and NLRs in a Solanum pangenome comprising chromosome-scale genomes. Snakemake was executed in a mamba v1.4.2 environment with snakemake v7.32.3 and cookiecutter v1.7.3.^20^ Genes were predicted de novo from the genome alone using Helixer v0.3.2 with the land_plant_v0.3_a_0080.h5 model.^23^ Protein sequences were extracted using AGAT v1.2.0 and used as input for Resistify.^24^ Ortholog analysis was conducted using OrthoFinder v2.5.5 using all predicted protein sequences.^25^ Transposable elements were annotated with the latest GitHub release of EDTA.^26^

Gene models were translated to bed format using AGAT v1.2.0 and overlaps with intact TEs were identified using bedtools v2.31.1 with the command bedtools intersect using the options -f 0.9 -wo.^27^ To identify previously characterised NLRs, protein sequences of a subset of *Solanum*-originating NLRs in the RefPlantNLR database were queried against each genome with blastp v2.15.0.^28^ The full pipeline and all post hoc analysis are available at https://github.com/SwiftSeal/pangenomics/.

To validate that LTR-embedded NLRs were not missed in the Helixer annotation of *C annuum*, all open reading frames of the TE annotations were searched for putative NLRs. Individual sequences were extracted with AGAT v1.2.0, from which open reading frames were identified and translated via orfipy v0.0.4, which was used as input for Resistify to identify putative NLRs.

## Results

### Performance against RefPlantNLR

**Figure 1.**
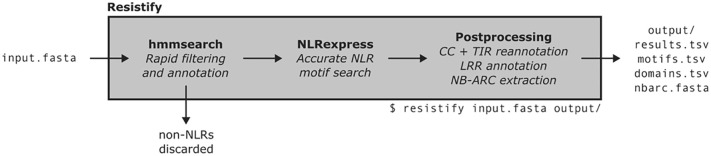
An overview of the Resistify programme, including input and output files, and command line usage.

To evaluate the performance of Resistify (Figure 1), it was applied against the RefPlantNLR database – a curated set of 415 previously cloned NLRs from a diverse range of species. NLRtracker was also applied to the RefPlantNLR database for comparison.

In the default mode, only 3 RefPlantNLR entries were not identified by Resistify – *AtNRG1.3* which carries only an LRR domain and *Pb1* and *RXL* which have significantly truncated NB-ARC domain that are not listed in the RefPlantNLR database. However, if required, these genes can be identified with Resistify using –ultra mode which skips the initial filtering stage and reports and sequence which contains at least one NLRexpress motif. Sequences are reported as an unmerged string of NLR motifs. Consequently, *AtNRG1.3*, *Pb1*, and *RXL* are reported as NNLLLLLLL, CNNNLLLLLLLLLLL, and CNNNLLLLLLLLL, respectively.

The largest source of variation between RefPlantNLR and Resistify classification was 29 NLRs which had an NL structure according to RefPlantNLR but CNL according to Resistify. All of these belonged to CNL-associated subclasses which are known to have CC domains that are challenging to identify. This included 15 CC_G10_-NLRs including *Pvr4*, *Tsw*, *RPS2*, *RPS5*, *SUT1*, and *SUMM2* which have previously been noted to lack a CC-associated EDVID motif.^29^ Others included 6 members of the *Pm5* locus which despite not containing a CC domain in RefPlantNLR, have previously been identified to contain CC-like domains.^30^ This analysis demonstrates that Resistify is highly sensitive at retrieving canonical NLRs and accurately describing their structure.

As NLRtracker does not exclude NLRs without NB-ARC domains, *AtNRG1.3*, *Pb1*, and *RXL* were identified and classified as ‘CC-NLR or CCR-NLR or CCG10-NLR’, ‘CC-NLR’, and ‘CC-NLR’, respectively. NLRtracker relies on the NB-ARC-associated RNBS-D motif to classify NLRs as ‘CC-NLR or CCR-NLR or CCG10-NLR’. As a result, 26 of the conflicting CNLs identified by Resistify were classified as ‘CC-NLR or CCR-NLR or CCG10-NLR’ despite failing to identify a CC domain. The exception of this was for *SpNBS-LRR*, *Rpp1-R1*, and *Rpp4C4* which were classified as ‘UNDETERMINED’.

Reliance on RNBS-D also led to classification of *NtTPN1* as ‘CC-NLR or CCR-NLR or CCG10-NLR’. While *NtTPN1* is a CNL ortholog, it lacks any upstream domain and is structurally an NL. Unexpectedly, the TNL *DSC1* was misclassified as ‘CC-NLR’ despite having a domain structure of ‘(TIR)(NBARC)(LRR)(CJID)’ according to NLRtracker.

In summary, Resistify performs well at identifying canonical NLRs from a diverse range of species. In default mode, it does not assign genes as NLRs with extremely truncated or entirely absent NB-ARC domains, unlike NLRtracker which reports any sequence with NLR-associated domains. However, this can be replicated in Resistify with the –ultra mode. Thus, Resistify’s structure-based classification method is well suited for correctly classifying NLRs, including members of challenging CNL subclasses.

### Performance against the Araport11 proteome

**Figure 2.**
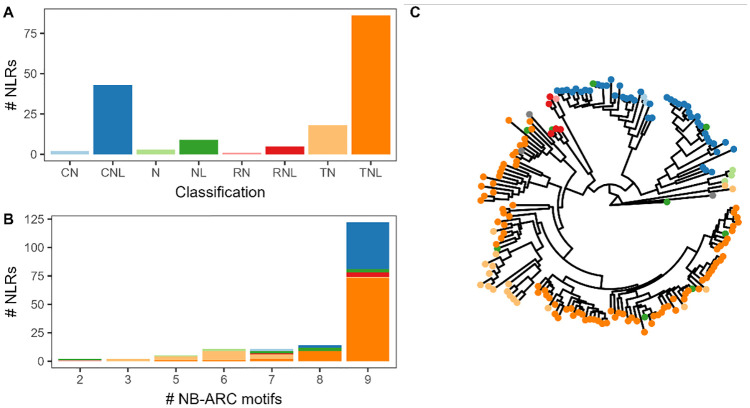
Resistify applied to Araport11. (A) The number of NLRs belonging to each identified classification by Resistify. (B) The number of NLRs grouped by number of identified unique NB-ARC-associated motifs coloured according to their classification. (C) A phylogenetic tree of NLRs based on the Resistify-extracted NB-ARC domain sequences, rooted to Ced-4. Tips are coloured according to their classification.

To assess the performance of Resistify across a well characterised and annotated genome, the representative gene models of Araport11 were analysed.^31^ In total, Resistify identified 166 NLRs – the majority of which were TNLs and CNLs (Figure 2A). Of the CNLs, 25% had a MADA motif, and 44.4% and 41.2% of NLs and TNLs had C-JID domain, respectively. Partial NLRs either without an N-terminal or LRR domain were also identified.

Manual inspection of the motifs within these sequences confirmed that they were not due to a failure to identify these elements. The majority of NLRs carried all 9 conserved NB-ARC motifs – those with fewer increasingly belonged to partial NLR classifications (Figure 2B). The NLRs with as few as 2 of the conserved NB-ARC motifs were successfully identified. A phylogenetic tree of the Resistify-identified NB-ARC domains independently validated the classifications and allowed the placement of ambiguous NL or N classified sequences into subclasses (Figure 2C).

NLRtracker identified an additional 48 sequences; however, these sequences did not contain any NB-ARC domain annotation according to either tool. Three sequences – AT4G19060.1, AT4G19060.1, and AT5G45440.1 – were not identified by NLRtracker. According to Resistify, these contained a single NB-ARC domain each, with 5, 6, and 6 NB-ARC motifs, respectively. Overall, Resistify performs well at identifying and classifying NLRs from whole proteomes and successfully retrieves NB-ARC domain annotations for phylogenetic analyses.

### Application against an example workflow

To demonstrate how Resistify might be implemented to identify novel resistance genes, a pangenome experiment was performed. Eighteen contiguous *Solanum* genomes were downloaded and processed with a simple workflow which predicts genes de novo, identifies orthologues, and classifies NLRs with Resistify.^32,33^ To obtain consistent gene annotations across the pangenome, the recently developed tool Helixer was selected for its near reference quality predictions and lack of requirement for repeat masking which is known to result in false-negative NLR annotations.^23,34^ The workflow also predicts TEs with EDTA. The *C annuum* genome was included not only as an outgroup, but also because recent analysis has suggested a substantial expansion of NLRs in this species associated with LTR TEs.^35^

Predicted gene content varied between genomes from 29 222 in *Solanum habrochaites* to 61 015 in the larger *C annuum* genome (Table S1). Transposable element content ranged from 55.2% in *Solanum neorickii* to 76.6% in *C annuum*. While there was no significant difference in total TE content between tuber-bearing and non-tuber-bearing genomes (*P* = .644), there was a significant increase in the number of intact TEs reported by EDTA in the genomes of tuber-bearing species (*P* = .008).

**Figure 3.**
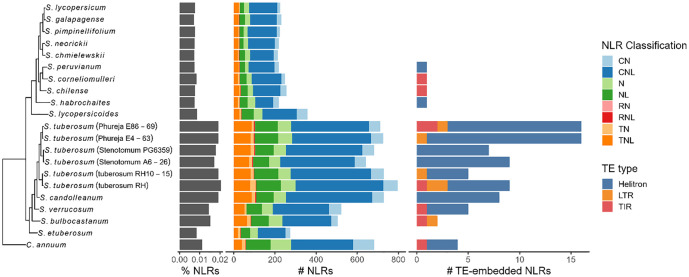
The phylogenetic tree of the Solanaceae pangenome calculated by OrthoFinder analysed in this study. The proportion of NLRs to total genes and the number of each NLR classification are highlighted for each genome. The number of NLRs embedded within intact transposable elements is also highlighted.

In total, 8144 NLRs were identified across all genomes (Figure 3). The CNLs were the most abundant classification of NLR identified, ranging from 84 in *S habrochaites* to 422 in *Solanum tuberosum* (group tuberosum RH10-15). There was a notable expansion of NLRs in tuber-bearing *Solanum* species. This is in agreement with the previous observation that potato-bearing *Solanum* species have an expansion of tuber expressed NLRs.^32^ A linear correlation between total NLR count and the proportion of genome occupied by TEs of any classification was not found (Figure S1).

**Figure 4.**
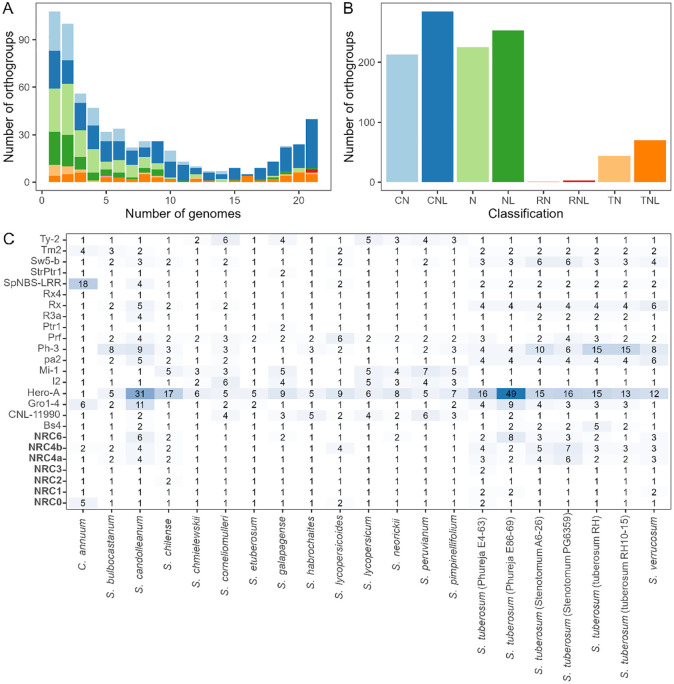
(A) The number of orthogroups shared between genomes in the pangenome. Orthogroups are coloured according to their majority classification. (B) The number of orthogroups according to NLR classification derived by Resistify. (C) The number of orthologs of known Solanaceae NLRs identified in each genome. The NRCs have been highlighted.

In total, 38 590 orthogroups were identified, of which 687 (1.8%) contained NLRs. The distribution of orthogroups showed a clear divide between core and shell/cloud orthogroups within the pangenome with most NLR orthogroups existing within the shell/cloud (Figure 4A). This reflects the relatively large width of the pangenome, which captures NLR variation over a genus level. Species-specific NLRomes also exhibit an abundance of cloud orthographs, reflecting the high variability of NLRs in genomes.^36^ Many (48.1%, n = 371) orthogroups were classified as containing N or NL NLRs (Figure 4B). While these classifications are less abundant (24.6%, n = 2081), they were often associated with scarce orthogroups contributing to this inflation (Figure 4A).

The distribution of previously identified Solanaceae NLR orthologs across the pangenome was examined (Figure 3). In agreement with previous findings, members of the NRC group remained relatively stable across the pangenome except for NRC4a, NRC4b, and NRC6 which were expanded in the tuber-bearing genomes. A general trend of ortholog expansion for known NLRs was seen in the tuber-bearing genomes. At least one ortholog was identified in each genome for all genomes.

It has previously been reported that there is a vast expansion of NLRs in the *C annuum* genome due to retroduplication; NLRs nested within LTRs represented a large proportion (~13%) of NLRs within the genome, and this effect is also seen in tomato (8%) and potato (18%).^35^ To explore whether this effect could be linked to the expansion of NLRs within the tuberising members of *Solanum*, a similar analysis was repeated. Intact TEs were identified and considered to interact with NLRs if they covered >90% of the gene annotation.

Unexpectedly, the effect could not be replicated and across all genomes only 5 intact LTRs were identified as containing NLRs (Figure 3). None were identified in *C annuum* but instead in the *S tuberosum* group Phureja, RH, and *Solanum bulbocastanum*. The putative retrotransposed NLRs within these all belonged to the same orthogroup which was expanded in both Phureja and RH, but not *Solanum stenotomum*. To verify that this was not due Helixer failing to predict NLRs contained within annotated LTRs, all open reading frames within all repeat annotations in the *C annuum* genome were searched for putative NLRs (Table S2). A total of 22 NLR-like sequences were identified – all except one were identified as only containing partial NB-ARC domains. In addition, none were identified within repeats classified as LTRs.

**Figure 5.**
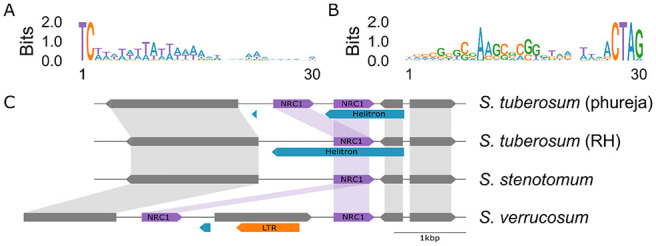
(A) The 5′ motif of NLR overlapping Helitrons. (B) The 3′ motif of NLR overlapping Helitrons. (C) The *NRC1* locus and its association with Helitrons across 4 genomes. Helitron (blue) and LTR (orange) transposable elements are highlighted.

By contrast, a proliferation of Helitron-associated NLRs was identified in the tuber-bearing genomes. All predicted Helitrons carried the expected 5′-TC. . .CTRR-3′ signature as well as a GC-rich region in the 3′ terminal (Figure 5A, B). An example of an NLR which may have undergone Helitron-associated duplication is *NRC1* (Figure 5C) which is present as a single copy except in both *S tuberosum* group Phureja genomes and *Solanum verrucosum*. Close examination of the *NRC1* locus reveals a duplication event in *S tuberosum* group Phureja where one *NRC1* gene is nested entirely within a predicted Helitron. In *S tuberosum* clone RH which only has one copy of *NRC1*, *NRC1* is similarly nested within a Helitron although this appears to be extended to a distant Helitron terminator past the site of duplication in group Phureja. In *S stenotomum*, only one *NRC1* is present and no Helitron predicted. Interestingly, *S verrucosum* has 2 copies of *NRC1*, and while it does not have a nested Helitron copy, the leftmost *NRC1* copy does have a short predicted Helitron upstream of its start position in the same orientation as the other Helitron elements. In this case, the 2 copies of *NRC1* appear to have been further separated by an intact LTR insertion which has resulted in an additional gene prediction.

## Discussion

Resistify is presented as a highly accurate and easy-to-use tool to aid the identification of novel resistance genes. Applying Resistify against the RefPlantNLR database demonstrates that it is effective at identifying NLRs from a diverse range of plant genomes. It is highly sensitive at classifying challenging CNL families which often evade other NLR classification tools due to the inclusion of the performant NLRexpress motif models. Members of the CC_G10_-NLR clade have previously been described as lacking the CC-associated EDVID motif and are often predicted as having an NL structure in the RefPlantNLR database.^29^ By utilising NLRexpress’ highly sensitive extended EDVID motif model, Resistify successfully classifies these elusive NLR clades, highlighting it as the most sensitive NLR classifier to date.

Resistify can be easily integrated into workflows and is scalable to large pangenome experiments. As high-quality genome assemblies become more common, providing accessible tools for the study of NLRs will be crucial for fully appreciating their diversity and identifying novel sources of resistance. The recent releases of chromosome-level assemblies for potato and tomato pangenomes are a valuable resource for understanding the diversity of NLRs within *Solanum*.^32,33^ An expansion of NLRs including essential families such as the NRCs is apparent in tuber-bearing species.

As Resistify relies on an initial NB-ARC domain search to reduce the search space prior to NLRexpress motif identification, NLRs with very truncated or entirely absent NB-ARC domains can be missed. To resolve this, an additional mode is provided which does not perform initial NB-ARC domain filtering which identifies sequences with any NLR motifs. Unlike NLRtracker, Resistify does not search for noncanonical integrated domains.^37^ As integrated domains are widespread and often critical to NLR function, their identification and analysis are an important factor in studies of NLR diversity.^3^ Pairing Resistify with a secondary sweep for these domains with NLRtracker or InterProScan would permit this analysis while also benefitting from a reduced search space and high-quality NLR classifications from Resistify.

An unexpected finding of this study was the failure to replicate the previous observation of an abundance of LTR-associated NLRs across Solanaceae.^35^ Differences in gene and TE annotation and NLR classification likely contributed to this. Although Helixer predicted ~70% more genes in *C annuum* than were identified in the previous study, this did not translate to an increase in NLRs identified. Instead, only 673 NLRs were identified here in comparison with the previous estimate of 835. Previously, a tBLASTn search of the NB-ARC domain followed by ORF identification and BLASTP searches against GenBank to classify NLRs was used to identify NLRs in the genome.^38^ This method may result in more false positives in comparison with identifying NLRs directly from gene annotations. Here, EDTA was selected for identifying TEs which has an improved sensitivity and selectivity for identifying intact LTRs in comparison with LTRHarvest which was used previously. Although Helixer does not require repeat masking which can result in false-negative NLR annotations, annotations used in training the model (which may have relied upon repeat masking) might introduce TE-avoidant behaviour.^34^ However, the identification of several LTR-associated NLRs indicates that this is unlikely to be the source of the difference in the result.

The identification of Helitron-associated NLR expansion has not been previously reported. Helitrons are challenging to identify due to their lack of structural elements, and as a result, the pipeline used in this study is known to overestimate Helitron density.^39^ However, a large part of this overestimation is likely due to EDTA reannotating the genome with HelitronScanner predictions, which itself suffers from a high false-positive rate, leading to a proliferation of fragmented Helitron annotations across the genome. For this study, only intact Helitrons which passed EDTAs stringent filter were considered. As a result, all predicted Helitrons had the required structural elements for activity. Further validation would be required to determine whether Helitron association is a valid mechanism of NLR expansion. The Helitron/*NRC1* relationship highlighted here would be a good starting point for unpicking this mechanism.

## Supplemental Material

sj-jpg-1-bbi-10.1177_11779322241308944 – Supplemental material for Resistify: A Novel NLR Classifier That Reveals Helitron-Associated NLR Expansion in Solanaceae Supplemental material, sj-jpg-1-bbi-10.1177_11779322241308944 for Resistify: A Novel NLR Classifier That Reveals Helitron-Associated NLR Expansion in Solanaceae by Moray Smith, John T Jones and Ingo Hein in Bioinformatics and Biology Insights

sj-xlsx-2-bbi-10.1177_11779322241308944 – Supplemental material for Resistify: A Novel NLR Classifier That Reveals Helitron-Associated NLR Expansion in Solanaceae Supplemental material, sj-xlsx-2-bbi-10.1177_11779322241308944 for Resistify: A Novel NLR Classifier That Reveals Helitron-Associated NLR Expansion in Solanaceae by Moray Smith, John T Jones and Ingo Hein in Bioinformatics and Biology Insights

sj-xlsx-3-bbi-10.1177_11779322241308944 – Supplemental material for Resistify: A Novel NLR Classifier That Reveals Helitron-Associated NLR Expansion in Solanaceae Supplemental material, sj-xlsx-3-bbi-10.1177_11779322241308944 for Resistify: A Novel NLR Classifier That Reveals Helitron-Associated NLR Expansion in Solanaceae by Moray Smith, John T Jones and Ingo Hein in Bioinformatics and Biology Insights
