# Supplementary figures and images for: Resistify: A Novel NLR Classifier That Reveals Helitron-Associated NLR Expansion in Solanaceae
Source: Bioinform Biol Insights. 2025 Jan 22;19:11779322241308944. doi: 10.1177/11779322241308944 (PMC11752215; doi:10.1177/11779322241308944)

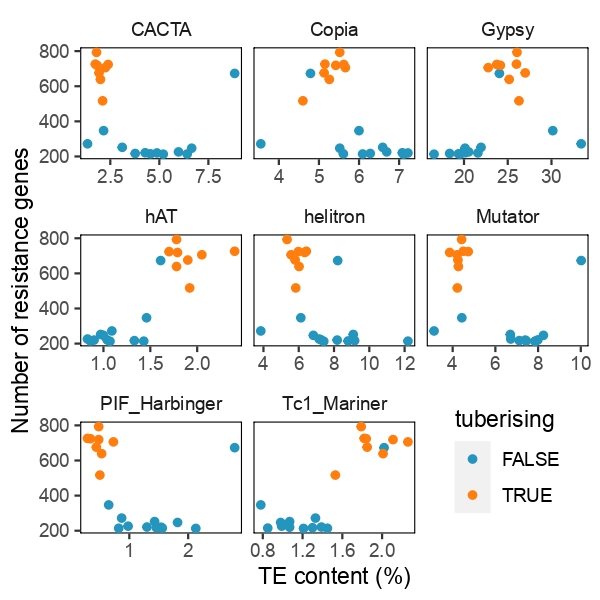

Supplement: sj-jpg-1-bbi-10.1177_11779322241308944 – Supplemental material for Resistify: A Novel NLR Classifier That Reveals Helitron-Associated NLR Expansion in Solanaceae [file sj-jpg-1-bbi-10.1177_11779322241308944.jpg]
